# Discrete-Event Simulation Thermal Model for Extrusion-Based Additive Manufacturing of PLA and ABS

**DOI:** 10.3390/ma13214985

**Published:** 2020-11-05

**Authors:** Sunil Bhandari, Roberto A. Lopez-Anido

**Affiliations:** 1 Advanced Structures and Composites Center, University of Maine, Orono, ME 04469, USA; rla@maine.edu; 2 Department of Civil and Environmental Engineering, University of Maine, Orono, ME 04469, USA

**Keywords:** fast thermal simulation, temperature-dependent properties, thermal history

## Abstract

The material properties of thermoplastic polymer parts manufactured by the extrusion-based additive manufacturing process are highly dependent on the thermal history. Different numerical models have been proposed to simulate the thermal history of a 3D-printed part. However, they are limited due to limited geometric applicability; low accuracy; or high computational demand. Can the time–temperature history of a 3D-printed part be simulated by a computationally less demanding, fast numerical model without losing accuracy? This paper describes the numerical implementation of a simplified discrete-event simulation model that offers accuracy comparable to a finite element model but is faster by two orders of magnitude. Two polymer systems with distinct thermal properties were selected to highlight differences in the simulation of the orthotropic response and the temperature-dependent material properties. The time–temperature histories from the numerical model were compared to the time–temperature histories from a conventional finite element model and were found to match closely. The proposed highly parallel numerical model was approximately 300–500 times faster in simulating thermal history compared to the conventional finite element model. The model would enable designers to compare the effects of several printing parameters for specific 3D-printed parts and select the most suitable parameters for the part.

## 1. Introduction

Extrusion-based additive manufacturing of thermoplastic polymers is a thermally driven process. Thermal history affects viscoelastic deformation [1,2,3], bonding [4,5,6,7,8], and residual stresses [9,10]. Consequently, dimensional accuracy and the strength of the manufactured part are driven by the thermal history of the part. 

Wang et al. [11] analyzed the strengths and weaknesses of different polymer-based additive processes including extrusion-based additive manufacturing. Tan et al. [12] reviewed the state of the art of commodity, engineering, and high-performance polymers used in additive manufacturing. Polymers undergo melting, thermal transitions, and solidification during the extrusion-based additive manufacturing process. Tan et al. [12] highlighted the different mechanical, rheological, and thermal properties that affect the additive manufacturing processability and the 3D-printed part properties. Several polymers have been successfully used in the extrusion-based additive manufacturing process. Initially amorphous polymers were preferred for additive manufacturing including acrylonitrile butadiene styrene (ABS) [13,14,15,16], polyethylene terephthalate glycol (PETG) [8,17], and polycarbonate (PC) [18,19,20]. The thermal and rheological properties of semi-crystalline polymers caused difficulties in filament production and showed higher shrinkage and warping during the 3D printing process [21,22]. Polylactic acid (PLA) [23,24,25,26], despite being a semi-crystalline polymer, is widely used in extrusion-based additive manufacturing, as the PLA crystallizes slowly and consequently shrinks and warps less than other typical semi-crystalline 3D-printed polymers. Recently, however, several semi-crystalline polymers such as polyamides (PA) [27,28], polyether ether ketone (PEEK) [29,30,31], and polypropylene (PP) [21,32,33] have been used for the extrusion-based 3D printing process. Suitable numerical models enable designers to account for the different properties of the polymers during the 3D printing process and optimize the design of 3D-printed parts [12,34,35,36].

Different numerical models have been proposed by researchers to simulate the thermal history of 3D-printed parts. Sun et al. [5] used a mathematical model to approximate the temperature of 3D-printed parts to model bond forming between acrylonitrile butadiene styrene (ABS) filaments. Wang et al. [37] used a simplified mathematical model to simulate the temperature and the subsequent warp deformation in extrusion-based 3D-printed ABS parts. These simplified layer-by-layer models have low accuracy and the results can only be interpreted qualitatively.

Compton et al. [38] discussed a 1D transient thermal model to describe a build process and analyze warping and cracking in thin-walled structures. The thermal model was solved by using a finite difference method that calculated the temperature at the nodes at each time step. Zhang et al. [39] used an adaptable, boundary adjusting finite difference method to simulate the thermal history of a 3D-printed polylactic acid (PLA) part. Stockman et al. [40] presented a thermal model tailored for additive manufacturing that was based on the 3D finite difference method. The researchers used coarse meshing in time and space along with simplifying assumptions about the solidification process. The finite difference scheme-based models work well for simple geometries such as thin-layered walls and rectangular cuboids. However, for more complex geometry parts that extrusion-based 3D printing usually produces, a method that can account for changes in geometry is necessary. 

Finite element analysis (FEA) modeling has been used to simulate the thermal history of 3D-printed parts with complex geometry. Ji and Zhou [41] used a finite element model that accounted for temperature-dependent material properties. D’Amico and Peterson [42] described an adaptable FEA model capable of simulating heat transfer in 3D and at sufficiently small time scales to capture rapid cooling. El Moumen et al. [43] discussed a 3D thermomechanical model that simulates the 3D printing process using FEA. Zhou et al. [44] described a finite element based on element activation to model the thermal history of a 3D-printed part. Zhou et al. [45] described a voxelization-based finite element simulation to simulate the thermal history of 3D-printed parts. Brenken et al. [3] used FEA modeling to simulate the thermal history, final deformed shape, and residual stresses in the 3D-printed short-carbon-fiber-reinforced ABS polymer. Finite difference methods and finite element methods solve systems of linear equations for each time step during the period of simulation. Such models slow down non-linearly as the size of the part to be simulated grows.

Heat flow through a body in the form of a partial differential equation is shown in Equation (1). Finite element methods and finite difference methods approximate a solution to the heat equations at discrete locations by solving a system of linear equations.

(1) $$\rho c_{p}\frac{\partial T}{\partial t}-\left(\frac{\partial }{\partial x}k_{x}\frac{\partial }{\partial x}+\frac{\partial }{\partial y}k_{y}\frac{\partial }{\partial y}+\frac{\partial }{\partial z}k_{z}\frac{\partial }{\partial z}\right)T=Q$$

where,

ρ = density;
$c_{p}\;$ = the specific heat capacity;
T = temperature;
$k_{x}$, $k_{y}$, and $k_{z}$ = conductivity in the x, y, and z directions, respectively;
Q = heat flow.

During 3D printing, the boundary of the part changes with each new deposition of beads. As a result, finite element methods and finite difference methods need to update the heat capacity matrix, the conductivity matrix, and the boundary conditions at each time step. Furthermore, non-linearities such as temperature-dependent conductivities and specific heat capacities require iterative solution of the system of linear equations at each time step. As a result, a system of linear equations-based solutions to the heat equation is slow and computationally expensive for simulating heat transfer during the 3D printing process. Some simplifications have been proposed for speeding up the simulations. McMillian et al. [46] reduced the 3D model to a 1D model by using geometric simplifications to create a computationally efficient finite difference method for metal additive manufacturing. Zhang and Shapiro [47] proposed a linear-time thermal simulation of as-manufactured fused deposition modeling components. They used the concept of “active body” to update the temperatures only in the elements that have been recently activated and using analytical equations to solve the temperature of “inactive” elements with “lazy updates”. The research work considered the thermal properties of ABS as constants for the simulation.

A phenomenological approach has been adopted in this paper to develop the thermal model for extrusion-based additive manufacturing. The phenomenological observations, are:

The geometry of the part changes rapidly during 3D printing. For desktop-scale printing on extrusion-based 3D printing, a bead deposition speed of 30–60 mm/s is commonly used [48]. It amounts to a material deposition rate of 30–60 cm^3^/h. For large-scale 3D printers, the deposition rates can reach 230 cm^3^/h [49]. The high rate of deposition continuously changes the geometry of the 3D-printed parts. 

Heat flow from one region of a part to another region of the same part is not instantaneous, i.e., heat transfer takes time. Polymers have low thermal diffusivity, which is a measure of the rapidity of heat propagation through a material [50]. 

The deposited element cools down quickly to reach the temperature of the surrounding environment (approximately 8 s for ABS 400 material [5,51]) and deposition in a distant layer has a negligible effect on the thermal history of an element. 

Based on the aforementioned observations, a solution to the heat transfer problem that examines local exchange of heat between neighboring regions at small time steps can be formulated. This research work treats deposition of a small bead of material as a discrete event. At small time steps, the deposited section of bead exchanges heat with its immediate neighbor via conduction, convection, or radiation. Temperature is considered static for the elements in a layer that are far away from the layer being actively deposited.

The objectives of the research presented herein are listed below. A model that meets these objectives would allow designers to quickly compare the effects of different printing parameters on the thermal history of a 3D-printed part and allow selecting the most suitable printing parameter for a given part.

Develop an efficient numerical model for thermal discrete-event simulation (DES) that generates the part geometry and mesh by interrogating the G-code, and uses parallel computation for fast analysis of extrusion-based additive manufacturing.

Simulate material orthotropy in 3D-printed parts by using information on the orientation of deposited beads from the G-code.

Capture the temperature-dependent response of the material in the simulation of the thermal behavior of 3D-printed polymers. 

Verify the accuracy of the proposed numerical model by comparing it with results from a finite element model using a commercial FEA package (Abaqus).

## 2. Materials and Methods 

### 2.1. Materials

PLA and ABS (3DXTech, Grand Rapids, MI, USA) were selected for this study. The polymers were chosen because they are widely used polymers used in the extrusion-based 3D printing process and represent two classes of polymers used in additive manufacturing, i.e., semi-crystalline and amorphous. PLA is a semi-crystalline material with a specific heat capacity that is dependent on temperature, with sharp changes at melting, cold crystallization, and glass transition temperatures [52,53]. ABS is an amorphous polymer with a fairly constant specific heat capacity [54]. Differential scanning calorimetry carried out during a previous study [8] was used for the specific heat capacity of PLA. 

### 2.2. Numerical Model

The following assumptions were used for the numerical model:

- Heat exchange occurs between neighboring elements only, for a small time step.
- The effect of radiation is not considered.
- Contact resistance is not considered in calculations involving thermal conductivity.

**Figure 1:**
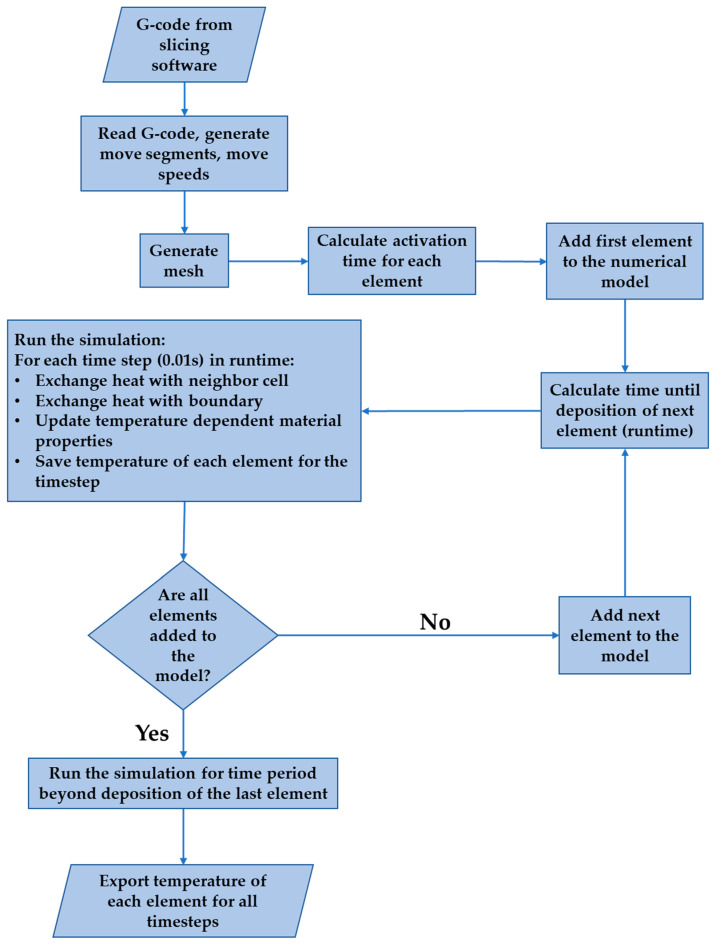
Flowchart of the numerical model used in this study.

Figure 1 shows the flowchart of the numerical model developed for this study. 

The model takes the G-code from the slicing software as input. The G-code is the sequence of machine instructions that directs the actions of the 3D printers. The G-code is generated by a slicing software using the geometrical model and the printing parameters as input. For this study, Simplify3D version 4.1.2 was used for slicing the geometrical model. Simplify3D was used because it is a widely used slicing software, enables modification of different printing parameters, and allows for the visualization of the printing process. The model reads the G-code and generates the movement segments, the speed of movement for each segment, and whether the extrusion is on for each segment. Table 1 shows the conversion of a section of the G-code output to a movement segment. X, Y and Z are the G-codes for the absolute position of the X, Y, and Z axes. G1 is the G-code instruction for linear interpolation. F is the G-code for setting extruder speed [55]. Each movement segment consists of an initial point, a final point, the movement speed, and information about whether extrusion occurs for the segment.

**Figure 2:**
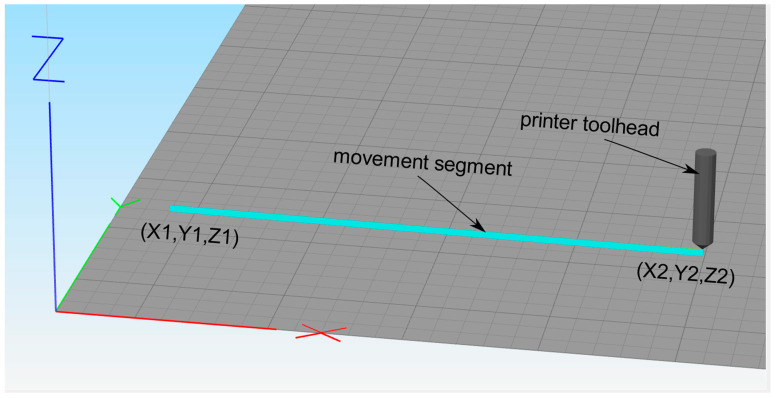
Movement segment coordinates with reference to machine axes.

In Table 1, X1, Y1, and Z1 are the coordinates of the initial point of the movement segment. X2, Y2, and Z2 are the coordinates of the final point of the movement segment. The coordinates are absolute coordinates with reference to the machine axes of the 3D printer. Figure 2 shows the movement segment with reference axis to the machine axes. The initial point of the segment is marked as (X1, Y1, Z1) and the final point of the segment, where the printer tool head is located, is marked as (X2, Y2, Z2).

**Figure 3:**
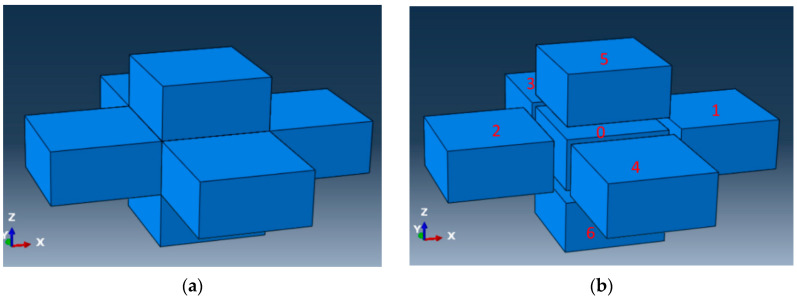
A configuration of elements showing neighbors. (**a**) elements on DES model and the neighbors; (**b**) exploded view of the elements and neighbors.

The mesh for the DES model described in this paper includes additional information that is not available in typical meshes used in finite element models. In finite element models, the mesh used has nodes defining the coordinates, and connectivity defining the elements. The mesh used for this study has, in addition to nodes and connectivity, the information about adjoining elements, called neighbor information for this study. For an element, the information about whether the faces are exposed to the boundary or connected to another element is the neighbor information for that element. For this study, hexahedral elements (brick elements) were considered with six faces. Figure 3 shows a configuration of elements in relation to their neighbors. Element 0 is hidden in Figure 3 on the left. Figure 3 on the right is an exploded view of Figure 3 on the left so that each element in the mesh can be viewed. For element 0, which is at the center, the neighbors at the front and the back are elements 1 and 2, respectively. Similarly, the neighbors at the right, left, top, and bottom are 4, 3, 5, and 6, respectively. Element 0 exchanges heat with elements 1, 2, 3, 4, 5, and 6 via conduction (and radiation if radiation is considered). Element 0 does not have any face exposed to the boundary and does not exchange heat with the environment via convection. Considering element 2, its only neighbor is element 0 at the front face. Element 2 exchanges heat with element 0 via conduction. Element 2 loses heat to the environment via convection (and radiation if radiation is considered) through the back, left, right, top, and bottom surfaces.

The neighbor information changes with each new element deposited and needs to be updated once an element is deposited.

**Figure 4:**
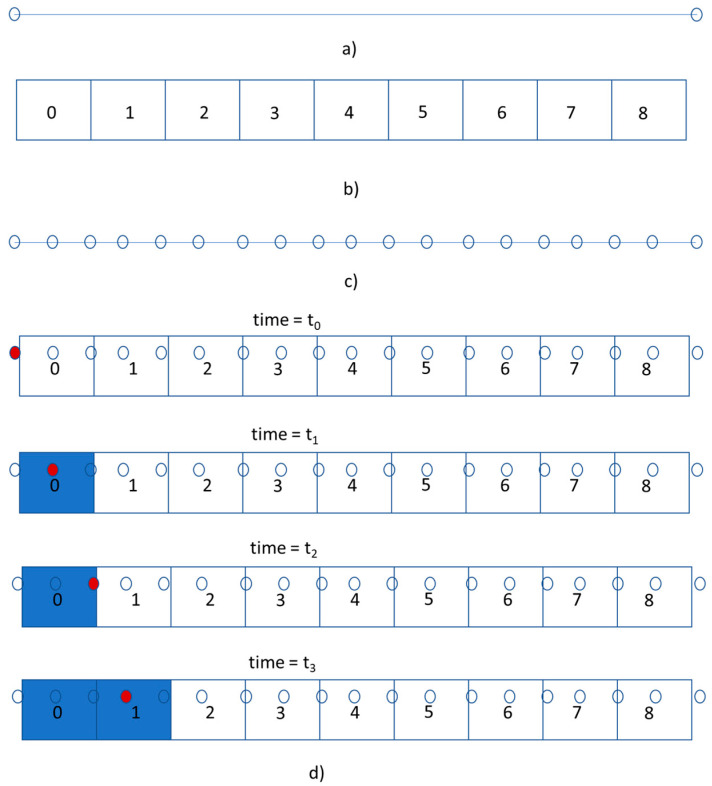
Calculation of the element deposition time and orientation. (**a**) movement segment generated from G-code; (**b**) top-view of hexahedral elements; (**c**) movement segment divided into points; (**d**) element deposition.

Based on the deposition path, the deposition time is generated for each element. Figure 4 shows the process for calculation of the element deposition time and the element orientation. Each movement segment is divided into points. The points mark the physical location of the deposition head during the 3D printing process. The points are created in a way that ensures that the spacing between two consecutive points is a length less than the length of an element. The time at which the movement head is at each point is calculated as t_p_. In Figure 4d, the red dots mark the movement of the deposition head. A square box with a width equal to the bead width, as shown in Figure 4d, is used to find elements that are deposited. If the center of the element is inside the square box, the element is considered deposited and the deposition time for the element is t_p_. The orientation of the element is calculated as the unit vector representing the movement segment. Element 0 is deposited at time t_0_, and element 1 is deposited at time t_3_.

The choice of the element dimensions is made such that the process of bead deposition can be simulated correctly. Each element has a length and width equal to the bead width of the 3D-printed part. The height of the element equals the bead height of the 3D-printed part. 

After the deposition time for all the elements have been calculated, the first element is added to the numerical model. The first element has a conductive boundary at the bottom face and a convective boundary at the front, back, right, left, and top faces. Until the time when the next element is deposited, for a time increment dt, heat losses to the environment due to convection are calculated using Equation (2).

(2) $$dQ=h\cdot A\cdot \left(T-T_{env}\right)\cdot dt$$

where,

dQ = heat loss to environment via convection,
h = convective heat transfer coefficient,
A = area of the surface exposed to the environment,
T = temperature at the centroid of the element,
$T_{env}$ = temperature of the environment, and
dt = time increment.

After adding the next element to the model, the neighbor information is updated for all the elements in the model. Conduction of heat from one element to the other is calculated using Equation (3).

(3) $$dQ=k\cdot A\cdot \frac{\left(T_{1}-T_{2}\right)}{dx}\cdot dt$$

where,

dQ = heat conducted to another element,
k = conductivity,
A = area of the conducting surface,
T = temperature at the centroid of the element,
$T_{env}$ = temperature of the environment,
dt = time increment.

The sum of heat exchanges through all surfaces and the new temperature of the element are calculated using Equations (3)–(6).

(4) $$dQ_{total}=\sum^{\;}dQ$$

(5) $$Q_{old}\;=\;T_{old}\cdot \rho \cdot C_{old}\cdot V$$

(6) $$Q_{new}=Q_{old}-dQ_{total}$$

(7) $$T_{new}=\frac{Q_{new}}{\rho \cdot C_{new}\cdot V}$$

where,

$dQ_{total}$ = total heat change from all surfaces,
$Q_{old}$ = total heat in the element during previous time step,
$T_{old}$ = temperature at the centroid of the element during the previous time step,
ρ = density of the element material,
C = the specific heat capacity of the element material at the old temperature,
V = volume of the element,
dt = time increment,
$Q_{new}$ = total heat in the element during this time step,
$T_{new}$ = temperature at the centroid of the element after current time step, and
$C_{new}$ = the specific heat capacity of the element after this time step.

Equation (7) is iterative and uses specific heat capacity versus temperature data for the element. Mass (volume times density) is assumed to be constant during the process.

Equation (8) shows the heat transferred through six different faces. The heat exchanged by an element with its neighbors is calculated using Equation (8). Equation (8) accounts for heat transferred via conduction. If any of the faces of the element is exposed to the environment, Equation (2) is used to account for heat exchange with the environment via convection for that face.

(8) $$\left\{ \begin{array}{c} dQ_{f}\\ dQ_{l}\\ dQ_{b}\\ dQ_{r}\\ dQ_{t}\\ dQ_{d} \end{array}\}=[\begin{array}{cccccc} k_{xx} & 0 & 0 & 0 & 0 & 0\\ 0 & k_{yy} & 0 & 0 & 0 & 0\\ 0 & 0 & k_{xx} & 0 & 0 & 0\\ 0 & 0 & 0 & k_{yy} & 0 & 0\\ 0 & 0 & 0 & 0 & k_{zz} & 0\\ 0 & 0 & 0 & 0 & 0 & k_{zz} \end{array}]\{\begin{array}{c} \frac{T-T_{f}}{dx}A_{f}\\ \frac{T-T_{l}}{dy}A_{l}\\ \frac{T-T_{b}}{dx}A_{b}\\ \frac{T-T_{r}}{dy}A_{r}\\ \frac{T-T_{t}}{dz}A_{t}\\ \frac{T-T_{d}}{dz}A_{d} \end{array}\}dt\right.$$

where,

$dQ_{f},\;dQ_{l},\;dQ_{b},\;dQ_{r},dQ_{t},\;\mathrm{and}\;dQ_{d}$ = heat exchange with the element connected to the front, left, back, right, top, and down faces, respectively;
$T_{f}$,$\;T_{l},\;T_{b},\;T_{r},\;T_{t},\;\mathrm{and}\;T_{d}$ = temperature of the element connected to the front, left, back, right, top, and down faces, respectively;
*dx*, *dy*, and *dz* = distance between the centroids of connected elements in the *x*, *y*, and *z* directions;
$A_{f},\;A_{l},A_{b},A_{r},A_{t},A_{d}$ = contact area with the element connected to the front, left, back, right, top, and down faces, respectively;
dt = time step.

Considering the front and back faces, the faces in the global x direction, and *A_b_* = *A_f_* = *A_x_*, *A*_l_ = *A_r_* = *A_y_*, *A_t_* = *A_d_* = *A_z_* for hexahedral elements used in this study, Equation (9) can be formed.

(9) $$dQ=dQ_{f}+dQ_{b}+dQ_{r}+dQ_{l}+dQ_{t}+dQ_{d\;}\;dQ=\left(k_{xx}\frac{T-T_{f}}{dx}A_{x}+k_{xx}\frac{T-T_{b}}{dx}A_{x}+k_{yy}\frac{T-T_{l}}{dy}y+k_{yy}\frac{T-T_{r}}{dy}A_{y}+k_{zz}\frac{T-T_{t}}{dz}A_{z}+k_{zz}\frac{T-T_{d}}{dx}A_{z}\right)dt\;dQ=\left(k_{xx}\left(\frac{-T_{b}+2T-T_{f}}{dx}\right)A_{x}+k_{yy}\left(\frac{-T_{l}+2T-T_{r}}{dy}\right)A_{y}+k_{zz}\left(\frac{-T_{t}+2T-T_{d}}{dz}\right)A_{z}\right)\;dt$$

Substituting Equation (9) in Equation (7), Equation (10) can be formed, which is equivalent to the forward time center space (FTCS) finite difference scheme for the Fourier heat transfer equation [56].

(10) $$\rho \cdot C_{new}\cdot V\cdot T_{new}\;=\;\rho \cdot C_{old}\cdot V\cdot T_{old}-\;\left(k_{xx}\left(\frac{-T_{b}+2T-T_{f}}{dx}\right)A_{x}+k_{yy}\left(\frac{-T_{l}+2T-T_{r}}{dy}\right)A_{y}+k_{zz}\left(\frac{-T_{t}+2T-T_{d}}{dz}\right)A_{z}\right)dt$$

**Figure 5:**
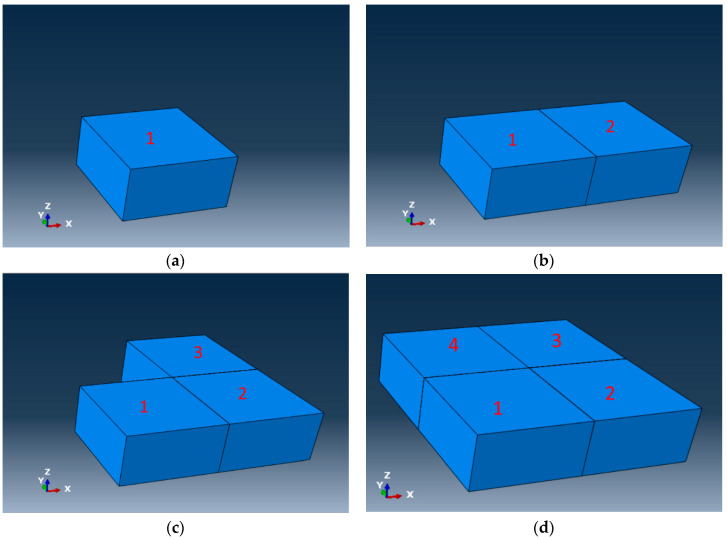
Changing neighbor information with addition of elements to the model. (**a**) *t* = *t*_1_, (**b**) *t* = *t*_2_, (**c**) *t* = *t*_3_, and (**d**) *t* = *t*_4__._

The temperature of each active element in the model is updated using the procedure described in Equations (2)–(7). When an element is added to the model, the neighbor information is updated to define the new boundary conditions. If an element is in a layer far away from the element just deposited, the far-away element is made inactive. Temperature is not updated for inactive elements in the model. For this study, a 20-layer distance was considered far, and elements more than 20 layers away from the element currently being deposited were made inactive. Figure 5 shows the changing neighbors with the addition of elements to the model. At time *t* = *t*_1_, element 1 has all faces exposed to the environment. At time *t* = *t*_2_, element 2 is added with neighbor element 1 at its back. The neighbor information of element 1 is also updated such that element 2 is at the front of the element 1. 

A stable time increment for a uniform grid in finite difference methods with forward Euler integration is given by Equation (11) [56].

(11) $$\Delta t<\left(\frac{\Delta x^{2}}{2\alpha }\right)$$

where,

α = $\frac{K}{\rho \cdot C}$ and
Δx = distance between the nodes.

Orthotropy in the conductivity of the 3D-printed material due to different conductivities along the bead and across the beads is considered. The conductivity matrix transformation from the local system to the global system is shown by Equation (12).

(12) $${\left[K\right]}_{G}={\left[T\right]}^{T}{\left[K\right]}_{l}\left[T\right]=\left[\begin{array}{ccc} c & -s & 0\\ s & c & 0\\ 0 & 0 & 1 \end{array}\right]\left[\begin{array}{ccc} k_{xx} & 0 & 0\\ 0 & k_{yy} & 0\\ 0 & 0 & k_{zz} \end{array}\right]\left[\begin{array}{ccc} c & s & 0\\ -s & c & 0\\ 0 & 0 & 1 \end{array}\right]=\left[\begin{array}{ccc} k_{xx}c^{2}+k_{yy}s^{2} & cs\left(k_{x}-k_{y}\right) & 0\\ cs\left(k_{x}-k_{y}\right) & k_{yy}c^{2}+k_{xx}s^{2} & 0\\ 0 & 0 & 1 \end{array}\right]$$

where,

${\left[K\right]}_{G}$ = the conductivity matrix in the global system (machine axes),
${\left[K\right]}_{l}$ = the conductivity matrix in the local system,
[T] = the transformation matrix,
*c* = cosine of bead deposition orientation to the machine X axis,
*s* = sine of bead deposition orientation to the machine X axis,
$k_{xx}$ = in-plane conductivity along the deposition of the bead,
$k_{yy}$ = in-plane conductivity perpendicular to the deposition of the bead, and
$k_{zz}$ = out-of-plane conductivity in the machine Z axis.

Equation (13) shows the heat transferred from the faces of an element via conduction for an orthotropic material.

(13) $$\left\{ \begin{array}{c} dQ_{f}\\ dQ_{l}\\ dQ_{b}\\ dQ_{r}\\ dQ_{t}\\ dQ_{d} \end{array}\}=[\begin{array}{cccccc} k_{xx}c^{2}+k_{yy}s^{2} & \frac{1}{2}cs\left(k_{x}-k_{y}\right) & 0 & \frac{1}{2}cs\left(k_{x}-k_{y}\right) & 0 & 0\\ \frac{1}{2}cs\left(k_{x}-k_{y}\right) & k_{xx}c^{2}+k_{yy}s^{2} & \frac{1}{2}cs\left(k_{x}-k_{y}\right) & 0 & 0 & 0\\ 0 & \frac{1}{2}cs\left(k_{x}-k_{y}\right) & k_{xx}c^{2}+k_{yy}s^{2} & \frac{1}{2}cs\left(k_{x}-k_{y}\right) & 0 & 0\\ \frac{1}{2}cs\left(k_{x}-k_{y}\right) & 0 & \frac{1}{2}cs\left(k_{x}-k_{y}\right) & k_{yy}c^{2}+k_{xx}s^{2} & 0 & 0\\ 0 & 0 & 0 & 0 & k_{zz} & 0\\ 0 & 0 & 0 & 0 & 0 & k_{zz} \end{array}]\{\begin{array}{c} \frac{T-T_{f}}{dx}A_{f}\\ \frac{T-T_{l}}{dy}A_{l}\\ \frac{T-T_{b}}{dx}A_{b}\\ \frac{T-T_{r}}{dy}A_{r}\\ \frac{T-T_{t}}{dz}A_{t}\\ \frac{T-T_{d}}{dz}A_{d} \end{array}\}dt\right.$$

The neighbor information is updated similarly for addition of element 3 and element 4, as shown in Table 2.

Once all the elements have been deposited, the model is allowed to run for a specified period of time to simulate the cooling down of the 3D-printed part. The time–temperature data for each element are exported to a file. The Rust programming language was used for discrete-event simulation due to its speed, automatic memory management without use of a garbage collector, and safe concurrency. Abaqus 2020 and its 3D printing module was used for thermal finite element modeling.

### 2.3. Part Geometry, Printing Parameters, G-Code Generation, and Inputs for the DES Model

**Figure 6:**
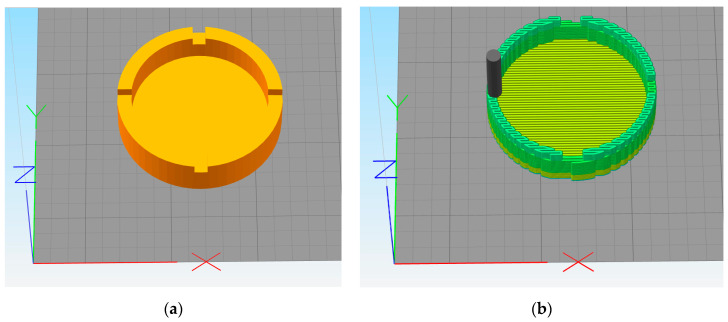
3D model and printing path for the 3D-printed part. (**a**) 3D model of the part, (**b**) Printing path for the part.

A solid modeling computer-aided design software (SolidWorks version 2016 SP5.0) was used for generating the geometry of the 3D-printed part. A miniature geometry ashtray model, as used by Zhou et al. [45], was used for this study. The geometry was considered complex enough to showcase the effects of 3D printing. The STL file was created using SolidWorks. Simplify3D software was used for slicing and generating the G-code tool path. Figure 6a shows the 3D model of the STL file imported into Simplify3D software. The part has a diameter of 48.8 mm and a height of 12.7 mm. Figure 6b shows the deposition path for the 3D-printed part.

The printing parameters used for this study are listed in Table 3.

A temperature of 25 °C was used as the environmental temperature. A convective heat transfer coefficient of 100 W·m^−2^·K^−1^ was assumed for this study. The thermal properties of PLA used for this study are listed in Table 4.

**Figure 7:**
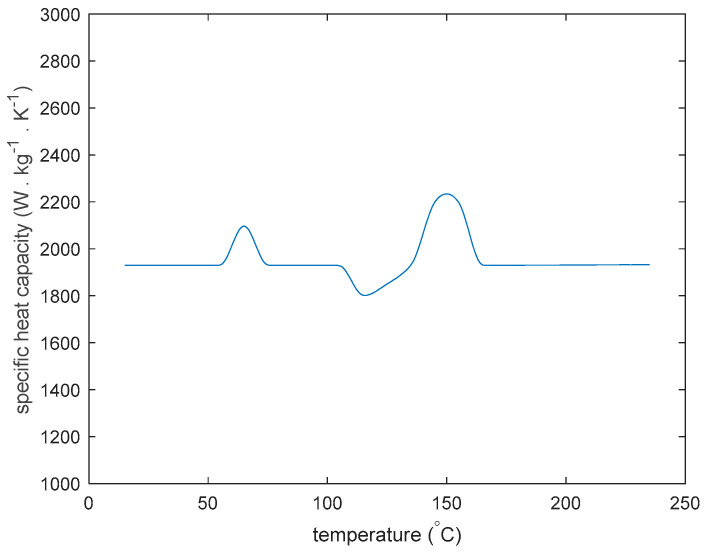
Specific heat capacity of PLA vs. temperature.

Specific heat capacities for PLA as a function of temperature are shown by the peaks and valleys in Figure 7. The specific heat capacity of PLA changes rapidly at the glass transition temperature at approximately 55 °C, at the cold crystallization temperature at approximately 120 °C, and at the melting point at approximately 150 °C.

Table 5 Thermal properties used for the ABS material during finite element analysis. The thermal properties are taken from the publication by Zhou et al. [45].

For a grid of 0.8 mm × 0.8 mm × 0.4 mm, with K = 0.20 W·m^−1^·K^−1^, ρ=1240 kg·m^−3^, and C=1400 J·kg^−1^·K^−1^, a stable time increment for PLA was calculated as 0.6944 s. 

For a grid of 0.8 mm × 0.8 mm × 0.4 mm, with K = 0.33 W·m^−1^·K^−1^, ρ=1050 kg·m^−3^, and C=780 J·kg^−1^·K^−1^, a stable time increment for ABS was calculated as 0.1985 s. 

For both cases, a time step of 0.01 s was chosen for the DES model. The maximum time increment that can be used for the DES model is the time required to deposit an element. The effect of smaller time increments on the results was studied by using four different time steps of 0.01, 0.005, 0.0025, and 0.00125 s. 

### 2.4. Finite Element Modeling Using Abaqus with the AM Modeler Plugin

Abaqus 2020 with the AM modeler plugin was used for the finite element analysis of the part. A cuboid with a length of 50.4 mm, a width of 50.4 mm, and a height of 13.2 mm was created. An 8-node hexahedral mesh (DC3D8) was created, where each element had a length and a width of 0.8 mm and a height of 0.4 mm. The width and height of the elements matched the width and height of the deposited beads. A total of 34071 elements were deposited in the final part during the analysis. A homogenous solid section property and material orientation directions matching the 3D printing direction were assigned to the part. The material properties used for the finite element analysis of PLA are listed in Table 4. A temperature of 25 °C was used as the environmental temperature. A convective heat transfer coefficient of 100 W·m^−2^·K^−1^ was assumed for this study. The thermal properties of PLA used for this study are listed in Table 4.

A transient thermal analysis was carried out with a time step of 1 s. Using a small time step would give a better resolution of temperature, capturing the peak temperatures better. However, the time required to complete the finite element simulation and the space required to store the output results increase with a decreasing time step. Increasing the time step would improve the speed of the simulation but miss capturing the peak and valley temperatures. A time step of 1 s was used as a compromise to obtain a good time–temperature resolution within a reasonable time period. A predefined temperature of 200 °C was used for PLA and a predefined temperature of 210 °C was used for ABS. 

The AM modeler plugin was used to define the progressive element activation and cooling interactions. A G-code to event series converter was written in the Rust programming language. The event series file generated was used as an input to the AM modeler plugin. An analysis job was set up with 6 processors enabled. 

Two elements, one deposited at the beginning of the simulation and another midway through the simulation, were chosen to compare the results from the FEA model to the results from the discrete-event simulation model. 

## 3. Results

**Figure 8:**
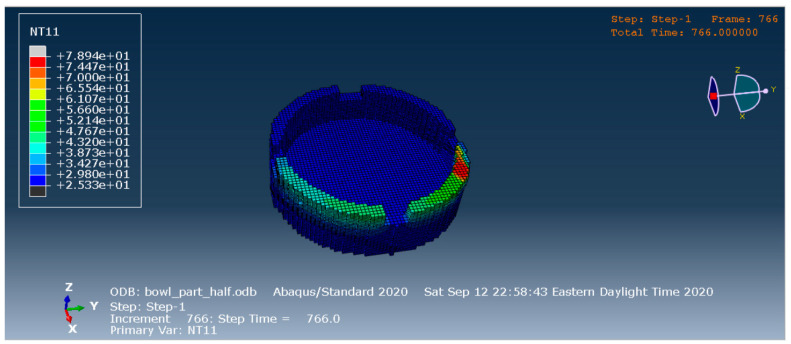
Temperature distribution in the model from FEA results.

Figure 8 shows the nodal temperature distribution in the part at 766 s from the beginning of the print for the finite element simulation. The finite element simulation ran until 778 s from the beginning of the print. The elements that are deposited later are hotter compared to the elements that were deposited earlier. 

**Figure 9:**
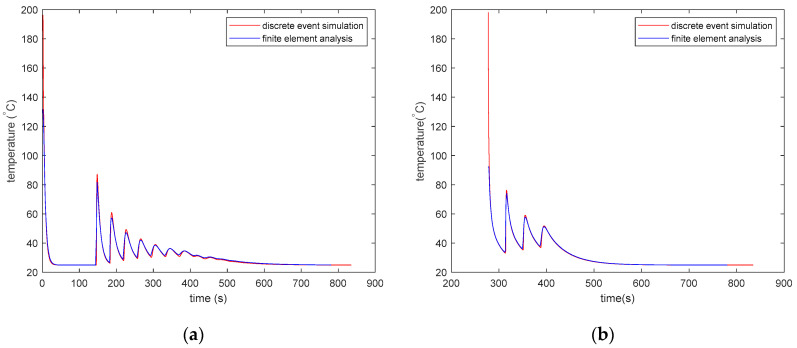
Comparison of the time–temperature history calculated using discrete element simulation and FEM for the PLA part. (**a**) The thermal history of the 34th deposited element with the centroid at (*x* = 26.0, *y* = 3.4, and *z* = 0.2 mm), and (**b**) the thermal history of the 12473th deposited element with the centroid at (*x* = 31.6, *y* = 28.2, and *z* = 1.8 mm).

Figure 9 shows the temperature versus time history of the 3D-printed part at two different locations for the PLA material. The thermal history obtained from the DES model closely matches that obtained from the FEA model. The DES model has a better resolution as it shows results from every 0.01 s. Hence, it can record the sharp peaks that arise when a new element is deposited next to another element. 

**Figure 10:**
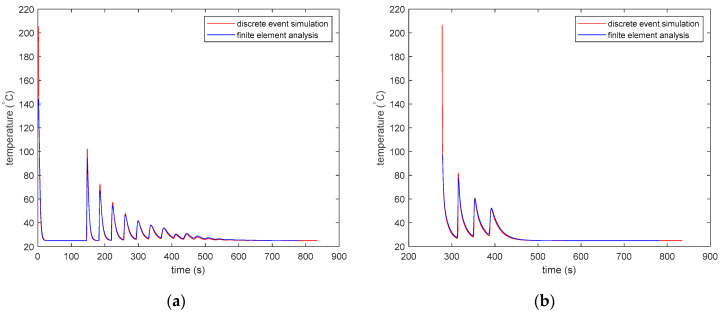
Comparison of the time–temperature history calculated using discrete element simulation and FEM for ABS part. (**a**) The thermal history of the 34th deposited element with the centroid at (*x* = 26.0, *y* = 3.4, and *z* = 0.2 mm), and (**b**) the thermal history of the 12473th deposited element with the centroid at (*x* = 31.6, *y* = 28.2, and *z* = 1.8 mm).

Figure 10 shows the temperature vs. time history of the 3D-printed part at two different locations for the ABS material. The thermal history obtained from the DES model closely matches the thermal history obtained from the FEA model. 

**Figure 11:**
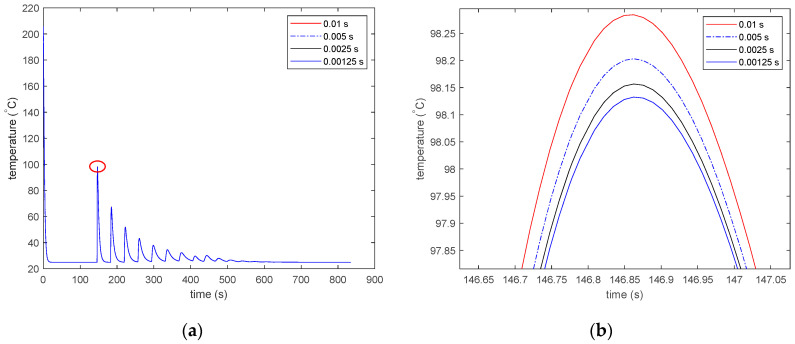
Effect of a decreasing time step on the DES model results for ABS. (**a**) The thermal history of the 34th deposited element with the centroid at (*x* = 26.0, *y* = 3.4, and *z* = 0.2 mm), and (**b**) the thermal history of the 34th deposited element zoomed in at the first reheating peak.

For both PLA and ABS, the time–temperature history starts recording after the first time step, which is 1s for the Abaqus FEA model and 0.01 s for the DES model. As a result, the initial temperature shown in the graphs is lower than the initial temperature of 200 °C for PLA and 210 °C for ABS. Figure 11 shows the effect of a decreasing time step on the results of the DES model. Figure 11a shows the thermal history of the 34th deposited element for the 3D-printed part with the ABS material. Four different time steps of 0.01, 0.005, 0.0025, and 0.00125 s are shown. However, the thermal histories obtained by using the four time steps are too close to see any difference in the line plots. The first reheating peak is marked with a red ellipse. The marked area is zoomed in and shown in Figure 11b to view the difference in results for models using the different time steps. The peak values seem to be converging with smaller time steps. With each halving of the time step, the difference in peak temperature becomes smaller. However, the difference between the peaks for models using a 0.00125 s time step and a 0.01 s time step is approximately 0.3 °C, which is very small compared to the temperature of the elements in the model.

**Figure 12:**
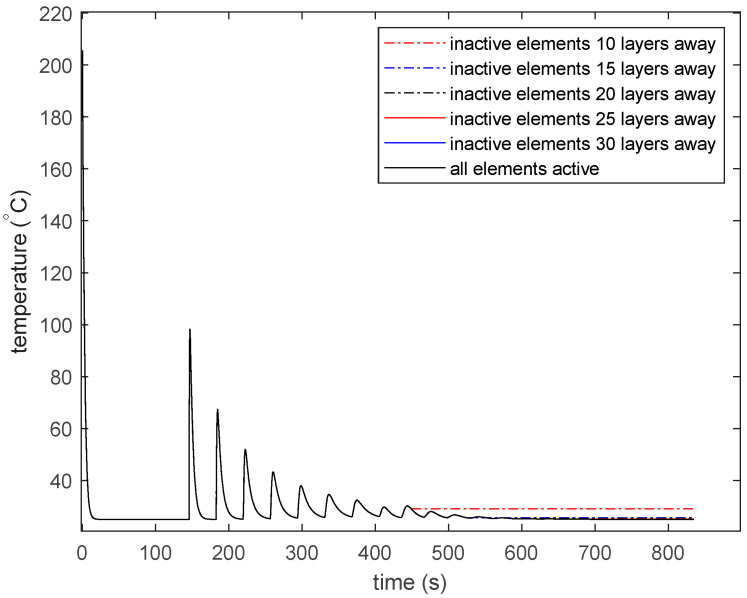
Effect of the distance of the inactive element from the most recently deposited layer.

Figure 12 shows the effect of making the 34th deposited element inactive when it is 10, 15, 20, 25, and 30 layers away from the most currently deposited layer. Figure 12 also shows the thermal history of the 34th deposited element when it is active throughout the analysis. The reheating peaks are approximately 4 °C high after 10 layers of deposition. If the element is made inactive after 10 layers of deposition, the reheating peaks that are lower than 4 °C will not be recorded by the model. Similarly, the reheating peaks after 15, 20, 25, and 30 layers of deposition are 0.6, 0.3, 0.08, and 0.01 °C high. The choice for distance to the currently deposited layer should be based on the accuracy desired for the model. Bonding between thermoplastic beads only happens at a temperature higher than the glass transition temperature [5,51]. Thermal models used to predict such bonding can use a 10-layer distance or lower for inactive elements. Young’s modulus for thermoplastic materials increases rapidly at the glass transition temperature with decreasing temperature. Models predicting residual stress are more sensitive to temperature changes at a lower temperature, as Young’s modulus for materials increases with decreasing temperature [43]. For such models that predict residual stresses, inactive elements that are 20 layers away might be necessary. 

The FEA modeling-based simulation for the PLA material was completed in 1 h 36 min (5766 s) with 6 cores. The simulation using the DES model was completed in 11.3 s with 6 cores. A speed up of 510 times was obtained using discrete-event simulation in this case.

The FEA modeling-based simulation for the ABS material was completed in 1 h 30 min (5420 s) with 6 cores. The simulation using the DES model was completed in 11.1 s with 6 cores. A speed up of 488 times was obtained using discrete-event simulation in this case.

**Figure 13:**
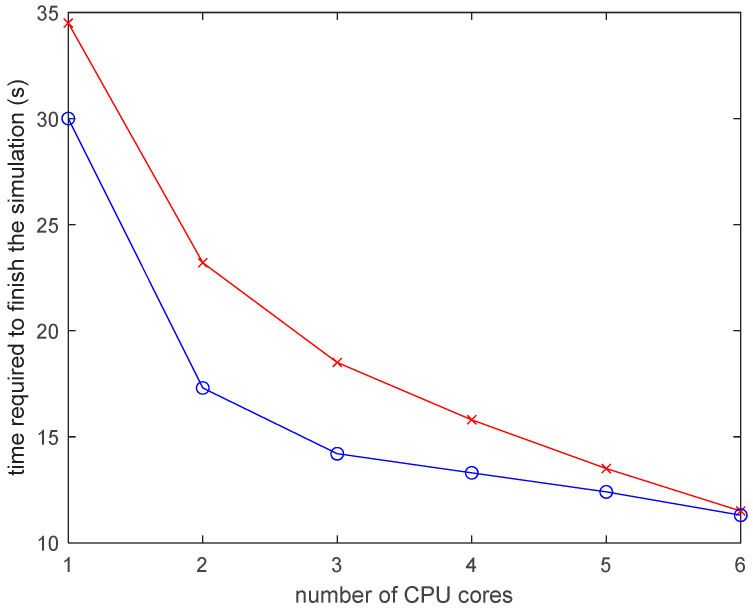
Time to finish the simulation vs. number of CPU cores used.

Figure 13 shows the speed up in the DES model by using a higher number of cores. The core of the algorithm used for calculating the temperatures is highly parallel. The temperature of an element is dependent only on its own properties and the properties of its immediate neighbors. The simulation can be sped up by using a higher number of CPU cores. 

## 4. Discussion

The experimental observations form different research works show certain features in the thermal history that could also be found in the thermal history presented by this model. It has been observed in various research works that the temperature of the deposited bead decreases rapidly after deposition [5,51]. The rapid cool down of the deposited element is due to the loss of heat to the neighboring bead due to conduction and the loss of heat to the environment due to convection. The heat loss is characterized by an exponentially decreasing temperature with time. The time–temperature history graph predicted by the model shows a similar temperature decrease in the deposited element. Another notable feature in the time–temperature history graph of the deposited beads in 3D printing is the rapid reheating by conduction of heat from the beads deposited above it [39,48,59,60]. The reheating of the elements is even more rapid than the cooling down of the element, with an almost instantaneous rise to the peak. The model presented in this manuscript shows similar reheating of deposited elements due to freshly deposited beads above it. A common observation in the thermal history of 3D-printed parts is the exponential decrease in reheating peaks with the increase in the distance to the newest layer deposited [47,51,59]. The model presented here shows a similar decrease in the reheating peaks, with depositions in layers 20 depositions away having a negligible effect on the thermal history of the 3D-printed part.

The DES model has a better resolution as it stores results from every 0.01 s. Hence, it can record the sharp peaks that arise when a new element is deposited next to another element. The difference in peaks between the FEA model and the DES model is higher for the PLA part than that for the ABS part. PLA exhibits much sharper changes in the specific heat capacity compared to ABS and a smaller time step is necessary to capture the changes in specific heat capacity of the PLA parts. However, the FEA model with a largertime step can only capture the changes in the specific heat capacity with a coarser resolution. 

The speed up results mainly due to two reasons. Firstly, the discrete element model only considers immediate neighbors for heat transfer for a small time step. As a result, solving a large system of linear equations is avoided. Furthermore, since only information about immediate neighbors is used for each element in a given time step, each element can be updated individually, independent of other elements that are not its immediate neighbors. As a result of this locality, parallel threads running on different cores can be used to update the temperature of each element for a given time step. Secondly, the model considers the elements in far-away layers (at least 20 layers away from layer being currently deposited for this study) to be inactive and does not update the temperature of such elements. Consequently, the number of elements that need to be updated at each time step is limited. As a result, the model can simulate the thermal history of 3D-printed material in linear time, i.e., increasing the model size increases the simulation time proportionally. In contrast, in conventional finite element models, increasing the size of the model increases the simulation time non-linearly, making the thermal simulation of 3D-printed large parts prohibitively long.

The numerical model presented is significantly faster than the equivalent finite element model. The DES model developed would allow designers to quickly compare the effects of different printing parameters on the thermal history of a 3D-printed part and allow the designers to select the most suitable printing parameters for a given part.

## 5. Conclusions

The following conclusions were drawn from the research work presented:

(1) A fast and accurate numerical model can be formulated based on heat exchange between deposited elements to generate the thermal history of a polymer extrusion-based 3D-printed part. The formulated DES numerical model was shown to be fast and accurate for the thermal simulation of extrusion-based additive manufacturing.
(2) Orthotropy in 3D-printed parts can be accounted for in the simplified numerical model by using the information on the orientation of deposited beads from the G-code. The effect of orthotropic conductivity in the 3D-printed PLA part was simulated using the DES model. The DES model was able to account for the orthotropic conductivity of the 3D-printed PLA part used for this study.
(3) The temperature-dependent material response can be captured in the DES model simulation of the thermal behavior of 3D-printed polymers. The temperature-dependent specific heat capacity was used for PLA and ABS. Temperature-dependent conductivities were used for ABS. The DES model can incorporate the effects of the temperature-dependent material properties of the 3D printing polymers.
(4) The accuracy of the DES model is comparable to the accuracy offered by finite element models. The results from the DES model were found to be comparable with the results from the FEA model.

## Figures and Tables

**Table 1:** Conversion from a G-code to a movement segment.

G-code	Movement Segment							
	X1 (mm)	Y1 (mm)	Z1 (mm)	X2 (mm)	Y2 (mm)	Z2 (mm)	Speed (mm/min)	Extrusion
G1 X0 Y0 F4800								
G1 Z0.400 F1002	0.000	0.000	0.000	0.000	0.000	0.400	1002	off
G1 X89.347 Y89.347 F4800	0.000	0.000	0.400	89.347	89.347	0.400	4800	off
G1 X110.653 Y89.347 E2.8347 F1300	89.347	89.347	0.400	110.653	89.347	0.400	1300	on
G1 X110.653 Y110.653 E2.8347	110.653	89.347	0.400	110.653	110.653	0.400	1300	on

**Table 2:** Neighbor information for cells with addition of new element at each time step.

Time Step	Elements	Front	Back	Right	Left	Top	Bottom
*t* = *t*_1_	1	env	env	env	env	env	env
*t* = *t*_2_	1	2	env	env	env	env	env
	2	env	1	env	env	env	env
*t* = *t*_3_	1	2	env	env	env	env	env
	2	env	1	env	3	env	env
	3	env	env	2	env	env	env
*t* = *t*_4_	1	2	env	env	4	env	env
	2	env	1	env	3	env	env
	3	env	4	2	env	env	env
	4	3	env	1	env	env	env

**Table 3:** Printing parameters used for this study.

Parameter	Value
Bead width	0.8 mm
Bead height	0.4 mm
Move speed	90 mm/s
Deposition speed	60 mm/s
Infill angle	0°
Deposition temperature	200 °C for PLA, 210 °C for ABS
Infill percentage	100%
First layer speed	12 mm/s

**Table 4:** Thermal properties used for the PLA material during finite element analysis.

Property	Value
density, ρ	1240 kg/m^3^ [57]
conductivity in the x direction, k_xx_	0.25 W·m^−1^·K^−1^ [58]
conductivity in the y direction, k_yy_	0.2 W·m^−1^·K^−1^
conductivity in the z direction, k_zz_	0.2 W·m^−1^·K^−1^

**Table 5:** Thermal properties used for the ABS material during finite element analysis [45].

Temperature (°C)	Thermal Conductivity (W·m^−1^·K^−1^)	Specific Heat Capacity (J·kg^−1^·K^−1^)	Density (kg·m^−3^)
0	0.23	780	1050
50	0.25	1040	1050
100	0.28	1490	1050
150	0.29	1710	1050
200	0.31	1865	1050
250	0.33	2020	1050
